# Dietary Oxysterol, 7-Ketocholesterol Accelerates Hepatic Lipid Accumulation and Macrophage Infiltration in Obese Mice

**DOI:** 10.3389/fendo.2020.614692

**Published:** 2021-03-10

**Authors:** Jiuyang Chang, Masahiro Koseki, Ayami Saga, Kotaro Kanno, Tomoaki Higo, Daisuke Okuzaki, Takeshi Okada, Hiroyasu Inui, Katsunao Tanaka, Masumi Asaji, Yinghong Zhu, Yoshihiro Kamada, Masafumi Ono, Toshiji Saibara, Ikuyo Ichi, Tohru Ohama, Makoto Nishida, Shizuya Yamashita, Yasushi Sakata

**Affiliations:** ^1^ Department of Cardiovascular Medicine, Osaka University Graduate School of Medicine, Osaka, Japan; ^2^ Genome Information Research Center, Research Institute for Microbial Diseases, Osaka University, Osaka, Japan; ^3^ Department of Molecular Biochemistry & Clinical Investigation, Osaka University Graduate School of Medicine, Osaka, Japan; ^4^ Division of Gastroenterology and Hepatology, Department of Internal Medicine, Tokyo Women’s Medical University Medical Center East, Tokyo, Japan; ^5^ Department of Gastroenterology and Hepatology, Kochi Medical School, Kochi, Japan; ^6^ Natural Science Division, Faculty of Core Research, Ochanomizu University, Tokyo, Japan; ^7^ Health Care Division, Health and Counseling Center, Osaka University, Osaka, Japan; ^8^ Department of Cardiology, Rinku General Medical Center, Osaka, Japan

**Keywords:** 7-ketocholesterol, steatohepatitis, obesity, macrophage, autophagy

## Abstract

Non-alcoholic fatty liver disease is strongly associated with obese and type 2 diabetes. It has been reported that an oxidized cholesterol, 7-ketocholesterol (7KC), might cause inflammatory response in macrophages and plasma 7KC concentration were higher in patients with cardiovascular diseases or diabetes. Therefore, we have decided to test whether small amount of 7KC in diet might induce hepatic steatosis and inflammation in two types of obese models. We found that addition of 0.01% 7KC either in chow diet (CD, regular chow diet with 1% cholesterol) or western type diet (WD, high fat diet with 1% cholesterol) accelerated hepatic neutral lipid accumulation by Oil Red O staining. Importantly, by lipid extraction analysis, it has been recognized that triglyceride rather than cholesterol species was significantly accumulated in CD+7KC compared to CD as well as in WD+7KC compared to WD. Immunostaining revealed that macrophages infiltration was increased in CD+7KC compared to CD, and also in WD+7KC compared to WD. These phenotypes were accompanied by inducing inflammatory response and downregulating fatty acid oxidation. Furthermore, RNA sequence analysis demonstrated that 7KC reduced expression of genes which related to autophagy process. Levels of LC3-II protein were decreased in WD+7KC compared to WD. Similarly, we have confirmed the effect of 7KC on acceleration of steatohepatitis in db/db mice model. Collectively, our study has demonstrated that small amount of dietary 7KC contributed to accelerate hepatic steatosis and inflammation in obese mice models.

## Introduction

Obesity is a complex disorder that has been a worldwide health problem for individuals as well as the society (1). The incident rate of Non-alcoholic fatty liver disease (NAFLD) is increasing and strongly associated with the patient’s background, such as obesity as well as type 2 diabetes mellitus (T2DM) (2–4). NAFLD could be categorized into non-alcoholic fatty liver (NAFL) with simple steatosis or non-alcoholic steatohepatitis (NASH) which is accompanied with steatosis, inflammation, and fibrosis (5). It is clinically important to distinguish two types of disease because the existence of inflammation could link to hepatic steatosis as well as systemic inflammatory disorders. Indeed, a couple of studies have demonstrated that the presence of NAFLD was associated with higher incident ratio of atherosclerotic cardiovascular diseases (CVD) (6, 7). Moreover, Yong-Ho and colleagues have demonstrated that hepatic steatosis was associated with left ventricular dysfunction (8). There is a growing evidence that diet can affect the pathophysiology of NAFLD as well as CVD (9–13).

An oxidized cholesterol, 7-ketocholesterol (7KC), can be produced by oxidation with oxygen, cooking, and reactive oxygen species (ROS) (14, 15). It has been reported that in our daily diet the concentration of 7KC was low compared to cholesterol (16), however, it might be unexpectedly increased by the advances in food manufacturing technology such as microwave cooking or long-term frozen storage (17). In *in vitro* experiments, it has been reported that 7KC has an ability to stimulate ROS production and eventually apoptosis due to cellular dysfunction in macrophages (18, 19). In an *in vivo* study, 7KC was toxic to macrophages through promoting inflammation in atherosclerotic lesions (20, 21). In human, it has been also reported that 7KC was detected in carotid atherosclerotic plaques (22). According to recent clinical studies, patients with higher blood 7KC concentration have a higher incidence rate of getting cardiovascular events (23, 24). Moreover, plasma 7KC levels were much higher in diabetes patients compared to healthy people (25, 26).

Considering these situations, it is quite precious to answer a question whether a small amount of 7KC in diet might affect the development of hepatic steatosis, inflammation, and fibrosis in obese mice to identify one of causal risk factors of steatohepatitis.

According to our results, the diet-derived 7KC accelerated hepatic steatosis and inflammation, without any change of lipid profiles or serum cytokines in obese mice models.

## Materials and Methods

### Animals and Diets

Male ob/ob or db/db mice were obtained from Charles River Laboratories (Tokyo, Japan) and housed in a temperature and humidity-controlled facility with a 12 h light/dark cycle. Ob/ob mice were fed regular chow diet with 1% cholesterol (CD, OrientalBio Laboratories, Chiba, Japan; casein 23%, sucrose 10%, corn oil 5%, and cholesterol 1%) with or without 0.01% 7KC and high fat, high cholesterol diet (WD, OrientalBio Laboratories, Chiba, Japan; casein 20%, sucrose 34%, cocoa butter 20%, and cholesterol 1%) with or without 7KC. Db/db mice were fed WD with or without 7KC. 7KC was purchased from Sigma-Aldrich (C2394, St. Louis, MO, USA).

Mice used for the experiment were anesthetized by an intraperitoneal injection of medetomidine (0.3 mg/kg), midazolam (4 mg/kg), and butorphanol (5 mg/kg). Adequate anesthesia was maintained by monitoring the respiration rate and the lack of response to paw pinching. The experimental protocol was approved by the Ethics Review Committee for Animal Experimentation of Osaka University Graduate School of Medicine.

### Biochemical Analyses

In serum, alanine aminotransferase (ALT), total cholesterol (TC), high density lipoprotein cholesterol (HDL-C), and triglycerides (TG) were measured by enzymatic methods (Wako Pure Chemical Industries, Tokyo, Japan). Non HDL-C was calculated as TC minus HDL-C. Hepatic TG, TC, and free cholesterol (FC) were also measured after lipid extraction of liver tissue by Folch method (Wako Pure Chemical Industries, Tokyo, Japan). Esterified cholesterol (CE) was calculated as TC minus FC. Serum tumor necrosis factor α (TNF-α) and IL-1β were determined using mouse TNF-α and IL-1β ELISA kit (MTA00B and MLB00C, Quantikine, Minneapolis, USA), respectively.

### Histologic and Immunohistochemical Analyses

Paraffin-embedded sections were stained with hematoxylin and eosin (200108, Muto Pure Chemicals, Tokyo, Japan) or sirius red (MKCB3138V, Sigma-Aldrich, Tokyo, Japan). For lipid staining, frozen sections were stained with Oil Red O (M3G0644, NACALAI TESQUE, Kyoto, Japan). Macrophages were detected by F4/80 (MCA497R, Bio-Rad, Tokyo, Japan) and VECTASTAIN secondary antibodies (Vector laboratories, Burlingame, USA). To quantify the area of staining by Oil Red O and Sirius Red, images of five random fields from each section were processed with Image J software (National Institute of Mental Health, Bethesda, MD, USA). Each value was expressed as the percentage of the total area of the section. Numbers of F4/80 positive cells were counted and averaged for five random fields of each section.

### Quantitative Polymerase Chain Reaction and Western Blotting

Quantitative real-time polymerase chain reaction (qRT-PCR) and western blot were performed as described previously (27, 28).

Briefly, total RNA was isolated from the liver tissues using the Rneasy^®^ Mini Kit (QIAGEN, Hilden, Germany). The RNA was reverse-transcribed using a SuperScript VILO cDNA Synthesis Kit (Thermo Fisher Scientific, CA, USA). qRT-PCR was performed by Taqman master mix (Thermo Fisher Scientific, CA, USA) and a 7900 Sequence Detection System (Applied Biosystems, USA). The specific primer information is listed in **Table S1**.

The antibodies used for the immunoblot are LC3B (2775, Cell Signaling Technology, Beverly, MA, USA) and Rubicon (8465, Cell Signaling Technology, Beverly, MA, USA). For signal normalization, anti-GAPDH antibody was used (MAB374, Millipore, Bedford, MA, USA). Membranes were imaged with the ImageQuant LAS 4000 camera system (GE Healthcare). The band intensity was quantified by Image J software.

### RNA-Seq Analysis

The quality of the RNA was assessed by spectrophotometer (NanoDrop 2000, Wilmington, USA). Sequencing was performed on an Illumina HiSeq 2500 platform in the 75-base single-end mode. The Illumina Casava 1.8.2 software was used for base-calling. The raw reads were mapped to the mouse reference genome sequences (mm10) using TopHat ver. 2.0.13 in combination with Bowtie2 ver. 2.2.3 and SAMtools ver. 0.1.19. The number of fragments per kilobase of exon per million mapped fragments (FPKMs) was calculated using Cufflinks ver. 2.2.143,44. Pathway analyses were conducted using a STRING network tool.

### Measurement of Mitochondrial Complex I Activity

Mitochondrial complex I enzyme activity was measured by Complex I Enzyme Activity Microplate Assay Kit (ab109721, Abcam, Cambridge, UK) according to the manufacturer protocol.

### Statistical Analyses

Results were shown as means ± SD. Comparisons between two groups were made by a two-tailed Student t test. For multiple group comparisons, a one-way ANOVA with a *post hoc* Tukey test was performed and P < 0.05 was considered statistically significant.

## Results

### The Addition of Small Amount of 7-Ketocholesterol in Diet Did Not Affect Body/Organ Weights, Alanine Aminotransferase, Tumor Necrosis Factor-α, or Lipid Profiles

**Figure 1:**
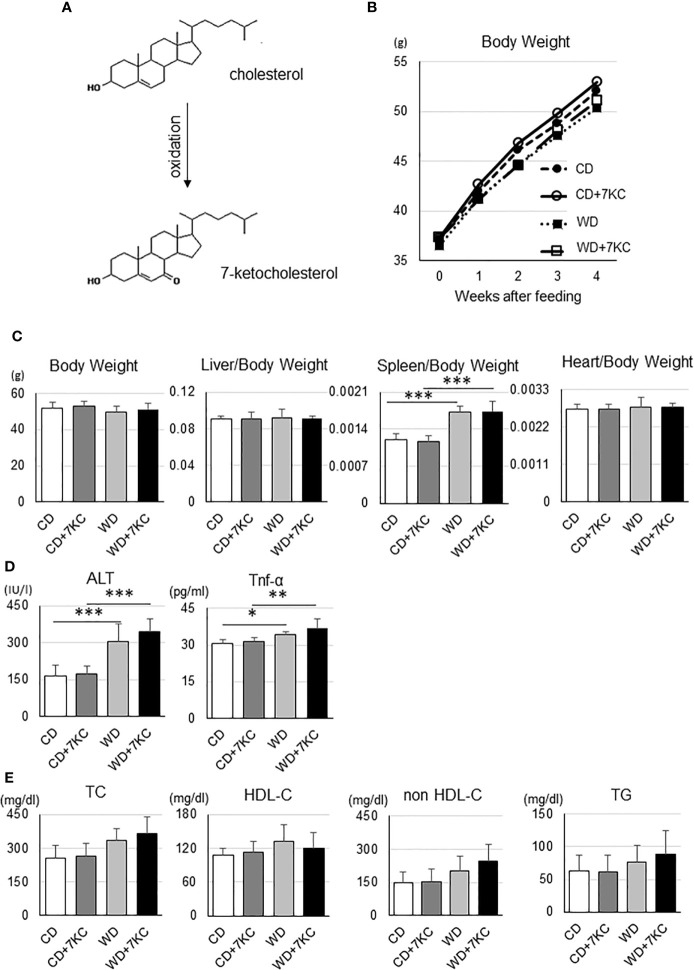
Effect of 7KC on body and organ weight, ALT, serum Tnf-α, and lipid profiles in ob/ob mice fed CD or WD for 4 weeks. **(A)** 7KC can be produced by oxidation from cholesterol, **(B)** The curve of body weight change with special diet feeding, **(C)** Body and organ weight, **(D)** Serum ALT and Tnf-α, **(E)** Lipid profiles. Plasma lipid concentrations were measured at the end of the feeding. All values are presented as the means ± SD. ANOVA with Tukey test, n = 6 per group; *p < 0.05, **p < 0.01, ***p < 0.001.

An oxidized cholesterol, 7KC, is recognized to be produced by oxidation from cholesterol (**Figure 1A**). In the current study, we would like to test whether small amount of 7KC supplementation in diet could accelerate hepatic lipid accumulation and inflammation in obese mice, we prepared four types of diets. As mentioned in *Introduction*, Ichi I et al. have investigated the daily intake of oxysterols and reported that cholesterol to 7KC was approximately 700 to 1. We have estimated that 7KC intake could be increased by five- to ten-fold due to increasing consumption of processed meat or sausage of microwave and finally determined that the ratio of cholesterol to 7KC should be 100 to 1. To make the ratio of cholesterol to 7KC 100:1, we increased cholesterol content of regular chow diet up to 1% (CD; casein 23%, sucrose 10%, corn oil 5%, and cholesterol 1%), with or without 0.01% 7KC. Also, high fat, high cholesterol diet (WD; casein 20%, sucrose 34%, cocoa butter 20%, and cholesterol 1%) with or without 7KC were prepared. Before we started the project, we have confirmed that CD+7KC or WD+7KC have not shown any liver injury in wild type/C57BL/6 mice even after 20 weeks (data not shown). Next, ob/ob mice at age of 6 weeks were fed CD, CD+7KC, WD, or WD+7KC for 4 weeks. Firstly, the change of body weight by 7KC was not observed both in CD and WD (**Figure 1B**). There were no difference of liver and heart weight in mice fed CD ± 7KC or WD ± 7KC (**Figure 1C**). Spleen weight of WD mice was significantly increased compared to CD (0.0017 ± 0.0001 *vs.* 0.0012 ± 0.0001; p < 0.001, **Figure 1C**), however, we could not see any difference between CD and CD+7KC or WD and WD+7KC. Similarly, in serum, alanine aminotransferase (ALT) level of WD mice was about two folds of CD (306.1 ± 68.8 *vs.* 163.5 ± 46.0; p < 0.001) and TNF-α level showed significantly higher than CD (34.1 ± 1.4 *vs.* 30.5 ± 1.9; p < 0.05), though the differences of ALT and TNF-α between CD and CD+7KC or WD and WD+7KC could not be observed (**Figure 1D**). There were no differences of total cholesterol (TC), high density lipoprotein cholesterol (HDL-C), non-HDL-C, or triglycerides in mice fed CD ± 7KC or WD ± 7KC (**Figure 1E**). These data suggested that the addition of small amount of 7-KC in diet did not affect body/organ weights, ALT, TNF-α, or lipid profiles.

### The Addition of Small Amount of 7-Ketocholesterol in Diet, However, Accelerated Hepatic Lipid Accumulation and Inflammatory Cell Infiltration in Ob/Ob Mice

**Figure 2:**
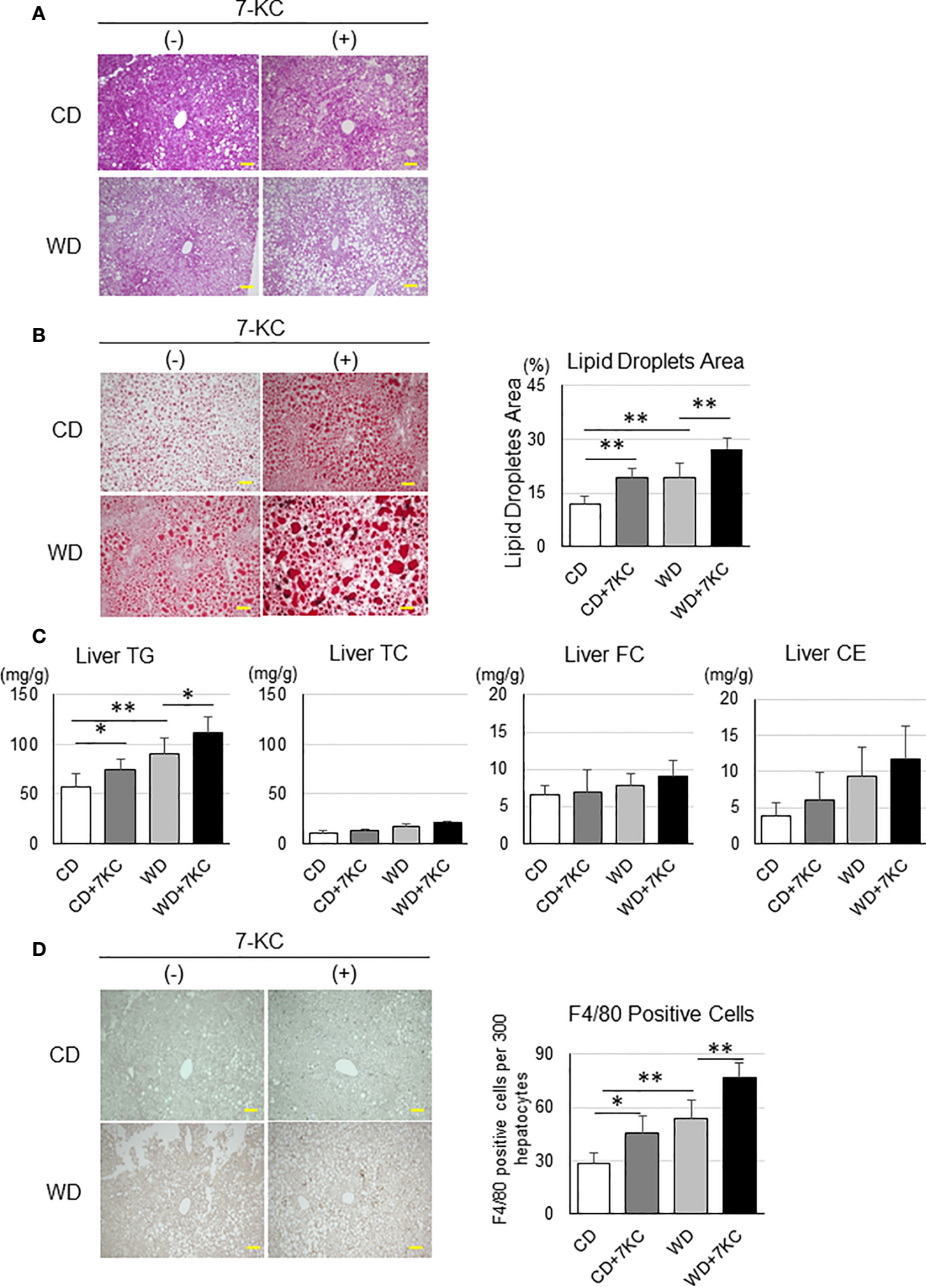
Addition of 7KC accelerated hepatic steatosis and inflammation in ob/ob mice fed CD or WD for 4 weeks. **(A)** HE staining in liver, **(B)** Oil Red O staining analysis, **(C)** hepatic lipid content, **(D)** F4/80 staining analysis. All values are presented as the means ± SD. ANOVA with Tukey test, n = 6 per group; Scale bars in **(A, B, D)** = 100 μm. *p < 0.05, **p < 0.01.

Serum ALT, TNF-α, and lipid profiles have never indicated the effect of 7KC, however, interestingly, liver sections have demonstrated dramatic changes. This is also a common problem in the patients with NAFLD. In many cases, it is quite difficult to distinguish the patients with NASH from NAFL by laboratory tests, such as liver function, lipid profiles, and inflammatory markers examination. In HE and Oil Red O staining, there were more and larger lipid droplets in CD+7KC compared to CD. The area of lipid droplets which was scanned by ImageJ software showed significantly increased (11.9 ± 2.3 *vs.* 19.1 ± 2.8; p < 0.01). In like manner, much more and larger lipid droplets could be observed in WD+7KC compared to WD. Lipid droplets area was significantly increased (19.5 ± 3.7 *vs.* 27.3 ± 3.2; p < 0.01, **Figures 2A, B**). Then, we addressed the question which kinds of lipids were accumulated by 7KC exposure. We extracted the lipids of liver and measured hepatic triglyceride (TG), total cholesterol (TC), free cholesterol (FC), and cholesterol ester (CE) contents. We found that hepatic TG content was significantly increased in CD+7KC compared to CD (57.0 ± 14.0 *vs.* 73.9 ± 10.9; p < 0.05). Similarly, 7KC significantly increased hepatic TG content in WD (90.7 ± 16.1 *vs.* 111.8 ± 16.5; p < 0.05). There were no differences of liver TC, FC, or CE in mice fed CD, CD+7KC, WD, or WD+7KC (**Figure 2C**). Thus, 7KC accelerated accumulation of TG but not cholesterol, suggesting that 7KC might alter fatty acids metabolism. Infiltration of F4/80-positive macrophages was increased in CD+7KC compared to CD (28.4 ± 6.3 *vs.* 45.2 ± 9.8; p < 0.05). In WD, 7KC could also increase the macrophage infiltration (53.4 ± 10.4 *vs.* 76.6 ± 8.3; p < 0.01, **Figure 2D**). In Sirius-red staining, the area of fibrosis was determined by ImageJ. It showed no difference in mice fed CD, CD+7KC, WD, or WD+7KC (**Supplementary Figure 1**).

### The Supplementation of Dietary 7-Ketocholesterol Induced Inflammatory Response and Downregulated Fatty Acid Oxidation

**Figure 3:**
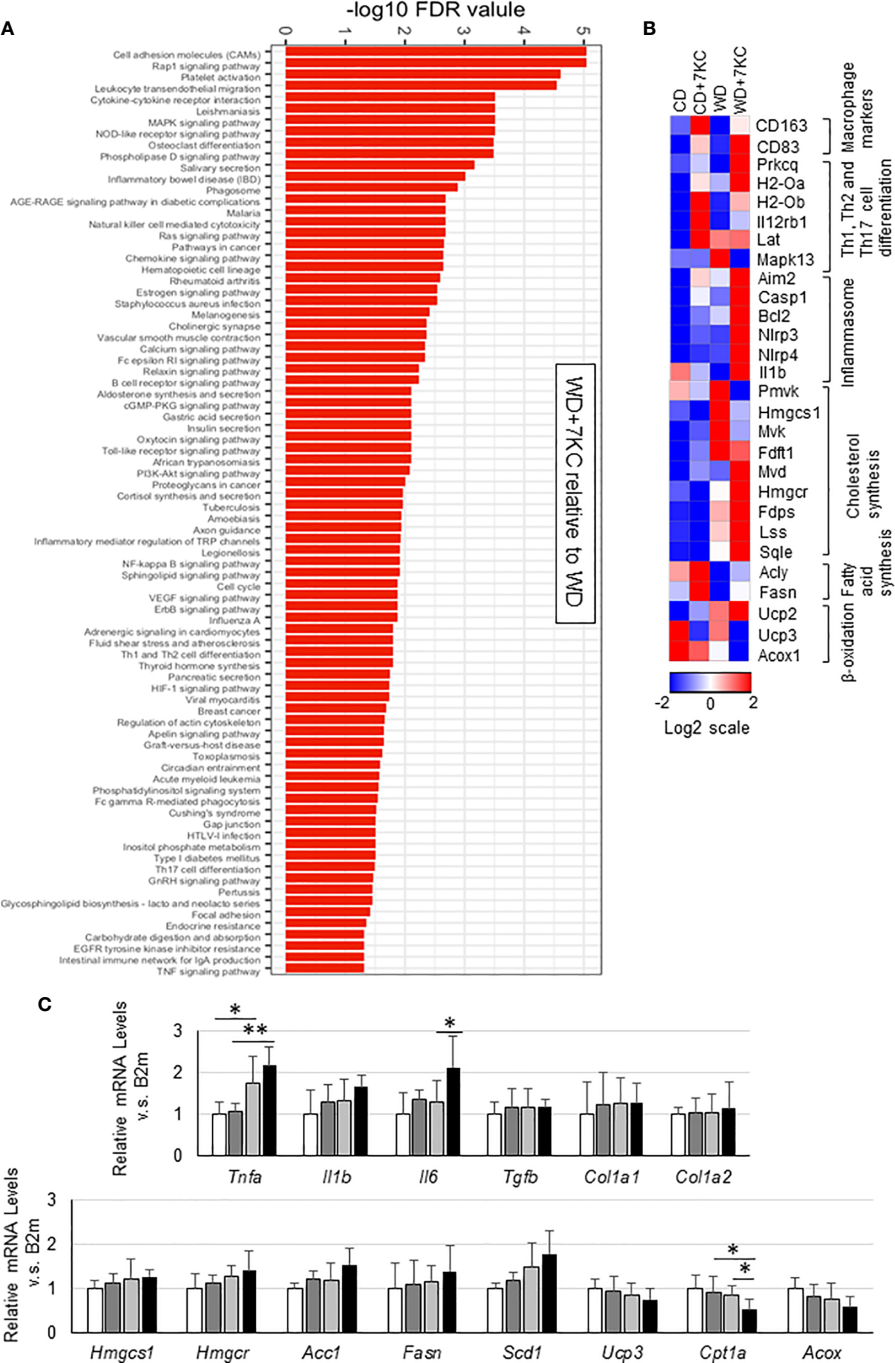
Hepatic gene expression in ob/ob mice fed CD or WD with or without 7KC for 4 weeks. **(A)** Pathways upregulated by addition of 7KC to WD, **(B)** Heatmaps from RNA-sequencing analysis, **(C)** mRNA expressions involved in inflammation and fibrosis, cholesterol synthesis, fatty acid synthesis, and β-oxidation. All values are presented as the means ± SD. ANOVA with Tukey test, n = 6 in **(C)** *p < 0.05, **p < 0.01.

To search the underlying molecular mechanism of accelerating hepatic lipid accumulation and inflammation, we analyzed hepatic mRNA expressions. To figure out the pathways enriched in WD+7KC, we performed RNA sequencing and put the genes whose log2 fold change was above 1 compared to WD diet into a STRING network tool. Addition of 7KC to WD upregulated a number of pathways, such as cell adhesion molecules, cytokine-receptor interaction, Ras signaling pathway, NF-kappa B signaling pathway, TNF signaling pathway, and so on (**Figure 3A**). We then examined each gene expression and found that 7KC upregulated most of macrophage maker genes and the genes related to Th1, Th2, and Th17 cell differentiation, inflammasomes, and cholesterol synthesis. Meanwhile, 7KC downregulated genes expression related to β-oxidation (**Figure 3B**). To confirm these expressions, we performed quantitative real-time PCR. Addition of 7KC in WD provoked a significant increase in mRNA expression of Interleukin 6 (IL-6) in ob/ob mice (1.3 ± 0.5 *vs.* 2.1 ± 0.7; p < 0.05, **Figure 3C**). TNF-α was significantly increased in WD compared to CD (1.0 ± 0.3 *vs.* 1.8 ± 0.6; p < 0.05, **Figure 3C**). However, there were no significant differences by addition of 7KC. Importantly, 7KC significantly decreased mRNA expression of *Cpt1a* in WD compared to WD+7KC (0.8 ± 0.2 *vs.* 0.5 ± 0.2; p < 0.05, **Figure 3C**) and mitochondrial complex I activity tended to decreased in WD+7KC (**Supplemental Figure 1A**). Collectively, these results suggested that 7KC induced inflammatory response and attenuated mitochondrial β-oxidation of fatty acids under triglycerides-overloading condition like WD+7KC, contributing to TG accumulation. In contrast, significant change of fibrosis gene expression could not be observed in ob/ob mice, which was consistent with the analysis of Sirius Red staining (**Figure 3C** and **Supplemental Figure 1B**).

### The Addition of 7-Ketocholesterol Suppressed Autophagy Process

**Figure 4:**
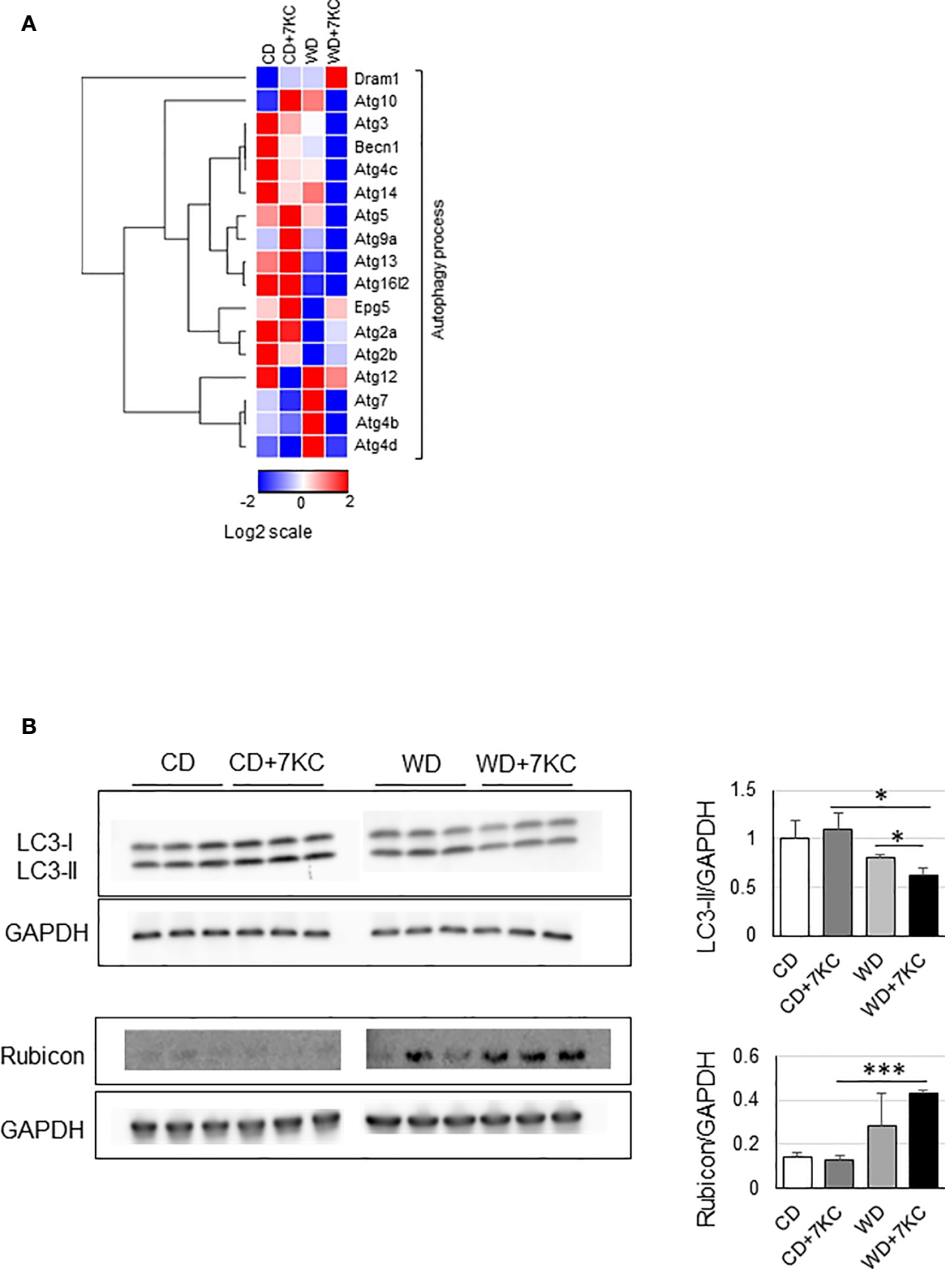
Effect of 7KC on autophagy in liver of ob/ob mice fed CD or WD for 4 weeks. **(A)** Heatmap of autophagy process-related genes, **(B)** Western bot for LC3-II and Rubicon. All values are presented as the means ± SD. ANOVA with Tukey test, n = 3 in B; *p < 0.05, ***p < 0.001.

To further explain the mechanism of 7KC accelerating steatohepatitis in ob/ob mice, we analyzed the mRNA and protein expressions involved in autophagy process since reduced autophagy in liver could result in marked hepatic steatosis (23). In WD+7KC, it showed decreased in the expression of genes related to autophagy process (Becn1, Atg3, Atg5, Atg7, Atg10, Atg13, and Atg14) (**Figure 4A**). Levels of LC3-II protein were decreased in WD+7KC compared to WD. Rubicon protein expression tended to increase in WD+7KC compared to WD (**Figure 4B**). These data suggested that 7KC suppressed autophagy process and this might be one of the reasons leading to severer steatohepatitis with obese.

### The addition of 7-Ketocholesterol accelerated steatohepatitis in Db/Db mice

**Figure 5:**
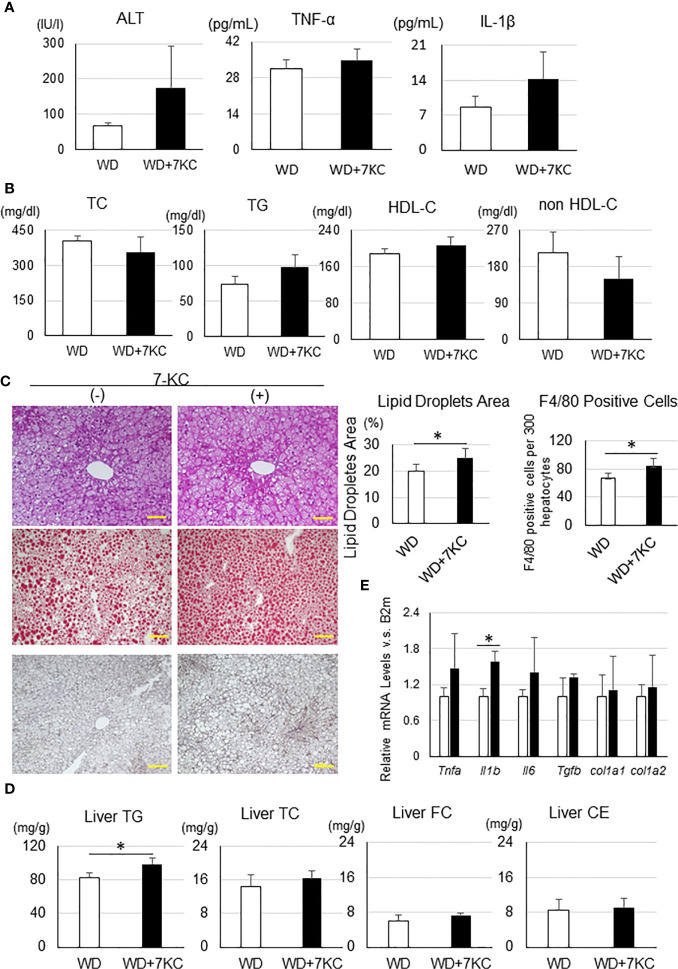
Effect of 7KC on body and organ weight, ALT, lipid profiles, liver histology, hepatic lipid content, and mRNA expression in db/db mice fed WD for 4 weeks. **(A)** Serum ALT, TNF-α, and IL-1β, **(B)** Lipid profiles, **(C)** HE staining, Oil Red O staining analysis, and F4/80 staining analysis, **(D)** mRNA expression, **(E)** Hepatic lipid analysis. All values are presented as the means ± SD. Student t test, n = 3 per group; *p < 0.05.

To determine whether 7KC exacerbates steatohepatitis in a T2DM obese model, we fed db/db mice with WD ± 7KC for 4 weeks. In serum, ALT, TNF-α, and IL-1β showed no differences with or without 7KC (**Figure 5A**). The change of lipid profiles could not be observed (**Figure 5B**). Liver histology showed same effect of 7KC with ob/ob mice. Hepatic steatosis and infiltration of macrophages were severer with addition of 7KC (**Figure 5C**). Liver TG content significantly increased in WD+7KC compared to WD (82.7 ± 5.5 *vs.* 98.3 ± 7.7; p < 0.05, **Figure 5D**). We analyzed hepatic mRNA expressions of genes involved in inflammatory and fibrosis in db/db mice. Interestingly, expression of IL-1β mRNA significantly increased in WD with 7KC (1.0 ± 0.1 *vs.* 1.6 ± 0.2; p < 0.05, **Figure 5E**).

### The Addition of 7-Ketocholesterol in Diet Did Not Affect Intestinal Lipid Absorption or Glucose Metabolism

To further investigate whether 7KC might have a primary effect on intestinal lipid absorption, lipid absorption test was performed (**Supplemental Figure 1C**). There were no differences of cholesterol and 7KC absorption between WD and WD+7KC. In addition, intraperitoneal glucose tolerance test (IPGTT) demonstrated no difference of glucose tolerance between WD and WD+7KC (**Supplemental Figure 2C**).

## Discussion

Oxysterols are categorized to two types, endogenous and exogenous sterols. Endogenous oxysterols such as 22-hydroxycholesterol and 27-hydroxycholesterol, are produced by endogenous enzymes when intracellular cholesterol excess, and act as a ligand for the nuclear receptor, liver X receptor (LXR), causing activation of a series of genes that carry cholesterol out of cells. Endogenous sterols are catabolized after acting as a ligand. On the other hand, exogenous sterols may be present in the diet and absorbed through NPC1L1 in intestine (29). Among the exogeneous oxysterols, those oxidized at the C7-position, such as 7KC, were detected in the atherosclerotic plaques (22). This could be explained by low expression level of ATP-binding cassette G1 (ABCG1) in macrophages, which can export 7KC from cells (30). Interestingly, ABCG1 mainly expresses in spleen, lung, and adrenal gland, but not in liver (30). Therefore, we hypothesized that 7KC may accelerate steatohepatitis similarly to CVD. Our results have clearly demonstrated that relatively small amount of dietary 7KC in which the ratio of cholesterol to 7KC was 100:1, accelerated hepatic lipid accumulation and macrophages infiltration in two types of obese mice. Importantly, addition of 7KC mainly increased hepatic TG, but not cholesterol. This could be explained by attenuating *Cpt1a* expression, represent of mitochondrial fatty acid β-oxidation, suggesting 7KC accumulation in organelles, especially mitochondria (31). Further investigation about membrane lipid composition on and in the cell will be required.

Regarding lipid content in NAFLD, fatty acids and TG were well investigated (32–34). These may be linked to the findings that low dietary sugar would effective in the patients with NAFLD (11, 12). In terms of cholesterol, it has been reported that desmosterol, a precursor of cholesterol, could predict steatohepatitis in human (35). Recently, Schnabel L. et al. have nicely demonstrated that ultra-processed food consumption would increase mortality (36). Because processed meat such as sausage and bacon are considered to contain relatively increased oxidized cholesterol, contribution of dietary 7KC in development of NAFLD could not be ignored and we should pay more attention to lipid quality in food.

As a causal risk factor of NAFLD, genetic background was well investigated. Previous genome-wide association study (GWAS) has revealed that I148M single nucleotide polymorphism (rs738409C> G) in patatin-like phospholipase domain containing 3 (PNPLA3) was detected as a susceptibility gene involved in the development of NAFLD/NASH (37, 38). However, these individuals with a minor allele has not shown higher incident ratio of CVD, suggesting that there might not be a genetic background sharing with NAFLD and CVD (9).

There are several limitations in this study. Although the importance of hepatic fibrosis in the point of mortality has been indicated (6, 39, 40), our analysis of Sirius Red staining and mRNA expression of fibrosis related genes could not indicate the effect of 7KC (**Supplementary Figure 1B**). Previous studies using ob/ob mice have shown that fibrosis was modest and that it was hardly aggravated by different factors. This resistance to fibrosis is attributable to the lack of leptin signaling which activates stellate cells and promotes fibrogenesis (41, 42). In addition, leptin has been demonstrated to promote inflammatory response with regulating the production of several cytokines like TNF-α and IL-6 (43). Thus, further investigations will be worth in another obese or diabetic model such as diet-induced obesity mice or streptozotocin-induced diabetic mice, to determine the effect of 7KC on hepatic fibrosis.

Collectively, we have proved that addition of 7KC in diet aggravated hepatic steatosis and inflammation without any change of body weight, serum lipid concentration and ALT level, suggesting the difficulties to diagnose the patients with NASH. A biomarker to distinguish NASH from NAFL such as Mac-2 binding protein is always valuable (44, 45). We would propose that dietary 7KC in diet could be an effective therapeutic target to reduce the burden of NAFLD/NASH and further investigation of serum 7KC as a biomarker for NAFLD/NASH will be expected.

## Data Availability Statement

The data presented in the study are deposited in the Gene Expression Omnibus (GEO) accession number GSE159234.

## Ethics Statement

The animal study was reviewed and approved by the Ethics Review Committee for Animal Experimentation of Osaka University Graduate School of Medicine.

## Author Contributions

JC and MK conceptualized the study. MK and YK developed the methodology. JC, AS, KK, DO, TOk, HI, KT, MA, and YZ conducted the investigation. TH and DO performed the RNA-Seq. JC and MK wrote and prepared the original draft. YK, MO, TS, II, TOh, MN, SY, and YS wrote, reviewed, and edited the manuscript. YS supervised the study. MK acquired the funding. All authors contributed to the article and approved the submitted version.

## Funding

This study was supported by a grant from the Program for Basic and Clinical Research on Hepatitis from Japan Agency for Medical Research and development (AMED) (Grant Number JP19fk0210031), JSPS KAKENHI grants (Grant Numbers 18H03532, 17H02100, 18K11020, 18K16026, 18H06222, 19K11766, 20K08383, and 20K17149) from the Japan Society for the Promotion of Science, and a grant from Project MEET, Osaka University Graduate School of Medicine/Mitsubishi Tanabe Pharma Corporation. The funder, Mitsubishi Tanabe Pharma Corporation, was not involved in the study design, collection, analysis, interpretation of data, the writing of this article or the decision to submit it for publication.

## Conflict of Interest

The authors declare that the research was conducted in the absence of any commercial or financial relationships that could be construed as a potential conflict of interest.

## Supplementary Material

Supplementary Figure 1 Effect of 7KC on complex I activity of liver and fibrosis in ob/ob mice fed CD or WD for 4 weeks. **(A)** Complex I activity of liver, **(B)** Sirius Red Staining and fibrosis area, **(C)** Intestinal lipid absorption. A bolus of 400 μl olive oil containing 0.4 μl [1,2-3H]-7KC (ART1174, Muromachi Kikai, Tokyo, Japan) and 2 μl [4-14C]-cholesterol (ARCO857, Muromachi Kikai, Tokyo, Japan) were orally administered to mice. Blood samples (50 μl) were drawn at 0, 2, 4, and 6 h from an orbital vein. Absorbed serum [3H]-7KC and [14C]-cholesterol were counted.

Supplementary Figure 2 Effect of 7KC on body and organ weight in db/db mice fed WD for 4 weeks. **(A)** The curve of body weight change with special diet feeding, **(B)** Body and organ weight. **(C)** IPGTT. The intraperitoneal injection of glucose (2 g/kg of body weight) in mice that had been fasted for 4 h and blood was obtained from a tail vein 30, 60, 90, or 120 min later.
