# Supplementary figures and images for: Dietary Oxysterol, 7-Ketocholesterol Accelerates Hepatic Lipid Accumulation and Macrophage Infiltration in Obese Mice
Source: Front Endocrinol (Lausanne). 2021 Mar 10;11:614692. doi: 10.3389/fendo.2020.614692 (PMC7989701; doi:10.3389/fendo.2020.614692)

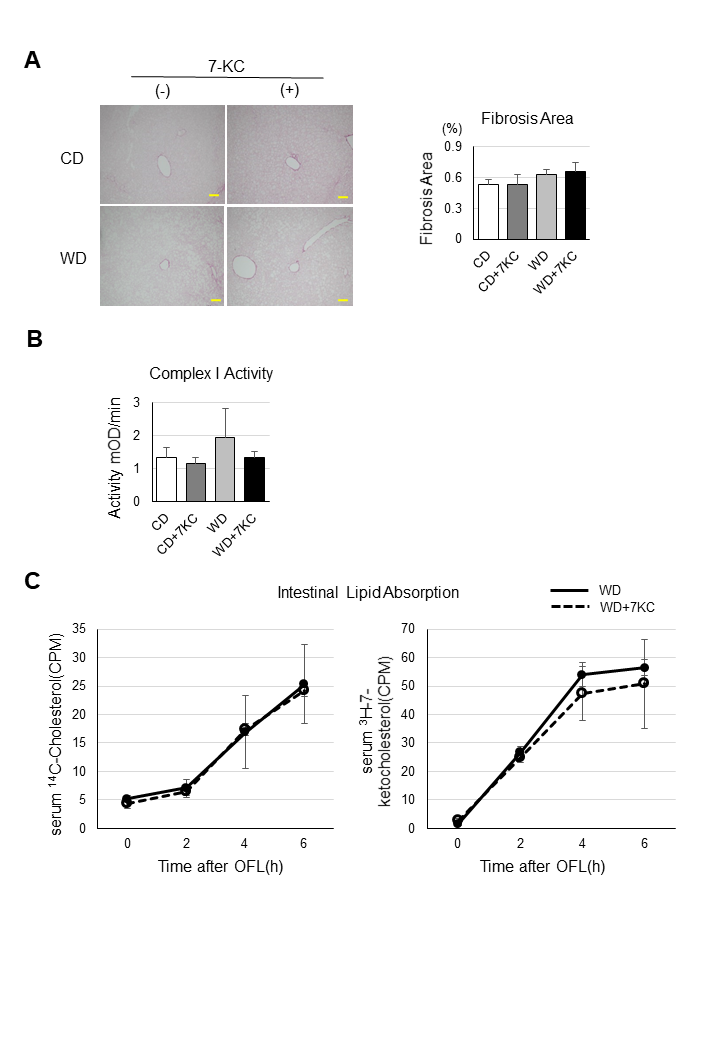

Supplement: Supplementary Figure 1 — Effect of 7KC on complex I activity of liver and fibrosis in ob/ob mice fed CD or WD for 4 weeks. (A) Complex I activity of liver, (B) Sirius Red Staining and fibrosis area, (C) Intestinal lipid absorption. A bolus of 400 μl olive oil containing 0.4 μl [1,2-3H]-7KC (ART1174, Muromachi Kikai, Tokyo, Japan) and 2 μl [4-14C]-cholesterol (ARCO857, Muromachi Kikai, Tokyo, Japan) were orally administered to mice. Blood samples (50 μl) were drawn at 0, 2, 4, and 6 h from an orbital vein. Absorbed serum [3H]-7KC and [14C]-cholesterol were counted. [file Image_1.tif]

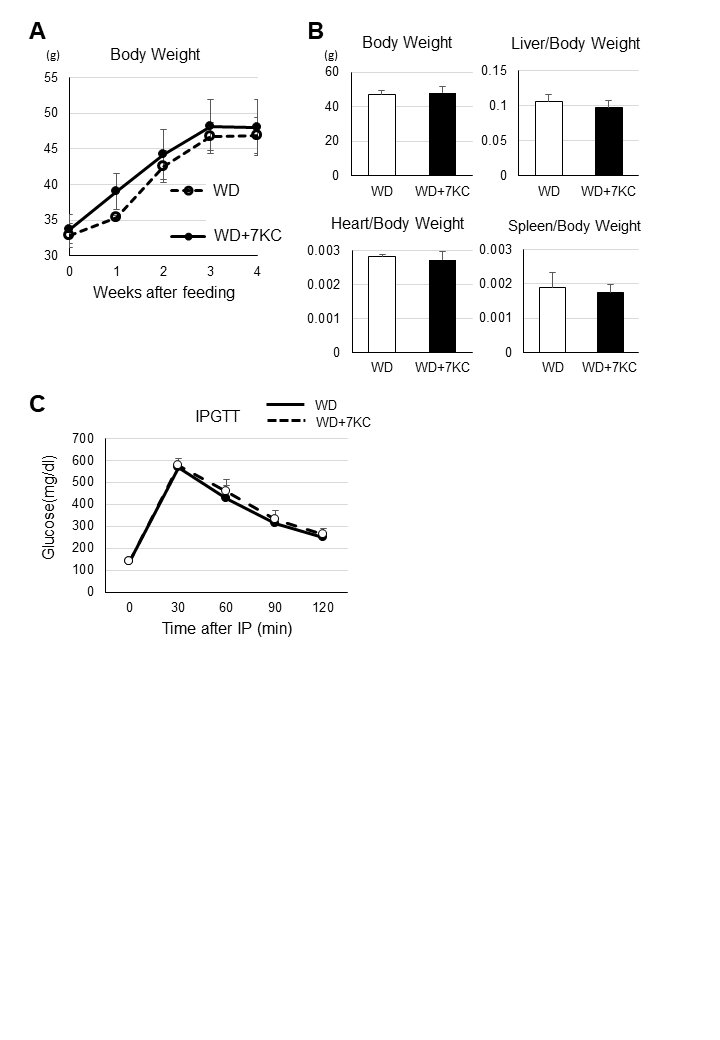

Supplement: Supplementary Figure 2 — Effect of 7KC on body and organ weight in db/db mice fed WD for 4 weeks. (A) The curve of body weight change with special diet feeding, (B) Body and organ weight. (C) IPGTT. The intraperitoneal injection of glucose (2 g/kg of body weight) in mice that had been fasted for 4 h and blood was obtained from a tail vein 30, 60, 90, or 120 min later. [file Image_2.tif]
