# Supplementary material for: Dietary Oxysterol, 7-Ketocholesterol Accelerates Hepatic Lipid Accumulation and Macrophage Infiltration in Obese Mice
Source: Front Endocrinol (Lausanne). 2021 Mar 10;11:614692. doi: 10.3389/fendo.2020.614692 (PMC7989701; doi:10.3389/fendo.2020.614692)
Supplement: Supplementary file 3 [file Table_1.docx]

**Supplemental Table S1. Primer list used for Real-time Quantitative RT-PCR.**

| TaqMan^®^ probes | |
| --- | --- |
| Tumor necrosis factor a (*Tnfa* ) | Mm00443258_m1 |
| Interleukin 1 beta (*Il1b*) | Mm00434228_m1 |
| Interleukin 6 (*Il6*) | Mm00446190m1 |
| Transforming growth factor beta (*Tgfb*) | Mm01178820_m1 |
| Collagen type I alpha 1 chain (*Col1a1*) | Mm00801666_m1 |
| Collagen type I alpha 2 chain (*Col1a2*) | Mm00483888 |
| 3-Hydroxy-3-Methylglutaryl-CoA Synthase 1 (*Hmgcs1*) | Mm01304569₋m1 |
| 3-hydroxy-3-methyl-glutaryl-CoA reductase (*Hmgr*) | Mm01282499_m1 |
| Acetyl-CoA carboxylase 1 (*Acc1*) | Mm01304257_m1 |
| Fatty acid synthase (*Fasn*) | Mm00662319_m1 |
| Stearoyl-CoA desaturase-1(Scd1) | Mm00772290_m1 |
| Uncoupling Protein 3 (*Ucp3*) | Mm01163394_m1 |
| Carnitine palmitoyltransferase Ia (*Cpt1a*) | Mm01231183_m1 |
| Acyl-CoA Oxidase 1 (Acox) | Mm01246835_m1 |
| Beta-2-Microglobulin (B2m) | Mm004377762₋m1 |
